# Deficiency of the Fanconi anemia E2 ubiqitin conjugase UBE2T only partially abrogates Alu-mediated recombination in a new model of homology dependent recombination

**DOI:** 10.1093/nar/gkz026

**Published:** 2019-02-01

**Authors:** Todd W Lewis, Joanna R Barthelemy, Elizabeth L Virts, Felicia M Kennedy, Rujuta Y Gadgil, Constanze Wiek, Rene M Linka, Feng Zhang, Paul R Andreassen, Helmut Hanenberg, Michael Leffak

**Affiliations:** 1 Department of Biochemistry and Molecular Biology, Boonshoft School of Medicine, Wright State University, Dayton, OH, USA; 2 Department of Pediatrics and Medical and Molecular Genetics, Indiana University School of Medicine, Indianapolis, IN 46202, USA; 3 Department of Otorhinolaryngology and Head/Neck Surgery, Heinrich Heine University, 40225 Duüsseldorf, Germany; 4 Division of Experimental Hematology & Cancer Biology, Cincinnati Children's Hospital Medical Center, Cincinnati, OH, USA; 5 Department of Pediatrics, University of Cincinnati College of Medicine, Cincinnati, OH, USA; 6 Department of Pediatrics III, University Children's Hospital Essen, University of Duisburg-Essen, 45122 Essen, Germany

## Abstract

The primary function of the UBE2T ubiquitin conjugase is in the monoubiquitination of the FANCI-FANCD2 heterodimer, a central step in the Fanconi anemia (FA) pathway. Genetic inactivation of *UBE2T* is responsible for the phenotypes of FANCT patients; however, a FANCT patient carrying a maternal duplication and a paternal deletion in the *UBE2T* loci displayed normal peripheral blood counts and UBE2T protein levels in B-lymphoblast cell lines. To test whether reversion by recombination between *UBE2T* AluYa5 elements could have occurred in the patient's hematopoietic stem cells despite the defects in homologous recombination (HR) in FA cells, we constructed HeLa cell lines containing the *UBE2T* AluYa5 elements and neighboring intervening sequences flanked by fluorescent reporter genes. Introduction of a DNA double strand break in the model *UBE2T* locus *in vivo* promoted single strand annealing (SSA) between proximal Alu elements and deletion of the intervening color marker gene, recapitulating the reversion of the *UBE2T* duplication in the FA patient. To test whether *UBE2T* null cells retain HR activity, the *UBE2T* genes were knocked out in HeLa cells and U2OS cells. CRISPR/Cas9-mediated genetic knockout of *UBE2T* only partially reduced HR, demonstrating that *UBE2T*-independent pathways can compensate for the recombination defect in *UBE2T/FANCT* null cells.

## INTRODUCTION

Alu elements are the most abundant short interspersed elements (SINEs) in the human genome, numbering over one million copies. These repetitive sequences are hotspots for genetic intrachromosomal or interchromosomal recombination (1). The proximity of abundant Alu elements in the genome clearly favors deletions by RAD51-independent intrachromosomal single strand annealing (SSA) (2). Alu-mediated recombination (AMR) events contribute to multiple forms of cancer and other genetic disorders (3–8), and are estimated to be responsible for 0.3% of human genetic diseases (4,9). These repeated elements also drive genomic evolution; it has been estimated that more than five hundred Alu-mediated deletion events have occurred since divergence of the human and chimpanzee genomes (9). Here, we modeled an unusual somatic reversion event in a Fanconi anemia (FA) patient who had inherited a partial genomic duplication in the *FANCT/UBE2T* gene from his mother. In the current model system, an *in vivo* double strand break leads to homology-dependent recombination between two *UBE2T* Alu elements, mimicking a contraction of the maternal duplication to restore the WT allele.

FA is a rare recessive or dominant DNA repair disorder characterized by genome instability, developmental abnormalities, bone marrow failure and cancer predisposition (10–12). Loss-of-function mutations in one X-chromosomal (*FANCB*) and at least twenty autosomal recessive genes (*FANCA* to *RFWD3/FANCW*) as well as missense mutations in one dominant negative FA gene (*RAD51A/FANCR*) result in the typical defects associated with FA (13–15). At the cellular level in FA deficient cells, genome instability in combination with erroneous repair of DNA interstrand crosslinks (ICLs) and DNA double strand breaks often results in complex genome rearrangements (CGR), translocations and gene amplification (16–18). Among the known activities of FA proteins are replisome stabilization during replication stress (17,19), the removal of DNA ICLs caused by endogenous aldehydes (20), the resolution of R-loops (21), stimulation of the alternative end joining (Alt-EJ)/microhomology-mediated end joining (MMEJ) of DNA double strand breaks (22), regulation of the spindle assembly checkpoint (23,24), and autophagic clearance of damaged mitochondria or viruses (25).

The diagnosis of FA is based on the combination of typical clinical symptoms and the characteristic hypersensitivity of cells from affected patients to the ICL reagents diepoxybutane (DEB), mitomycin C (MMC), melphalan or cisplatin, which often are used to dissect the functions of individual FA proteins (18,26). A key step in activating ICL repair is the monoubiquitination of FANCI and FANCD2 in the FANCI-FANCD2 (ID2) protein complex by the thirteen subunit FA core complex containing FANCL as the E3 ubiquitin ligase (reviewed in (26,27)). The *FANCT/UBE2T* gene product is not part of this protein complex but encodes the major E2 ubiquitin conjugating enzyme used by the FANCL E3 ligase to modify and activate the DNA-bound ID2 dimer (28–31). Monoubiquitination of FANCI and FANCD2 is necessary for their co-localization into nuclear foci. Additional roles for FANCI and FANCD2 in the stabilization of replication forks and HR have also been reported (17,30,32–35).

Machida *et al.* (36) and Alpi *et al.* (37) have shown that UBE2T is the E2 conjugating ligase in the FA pathway and that genetic deficiency in *UBE2T*^−/−^ DT40 cells leads to the classical cellular phenotypes of FA, including hypersensitivity to low doses of DNA ICL agents and high frequencies of chromosomal abnormalities. Subsequently, three groups including ours independently described three FA patients with germ-line defects in the *UBE2T* gene, now also designated *FANCT* (18,38–40). The 16-year-old FA patient (100166/1) of Italian ancestry described by us (40) was born with bilateral malformations of both thumbs and radii, microcephaly, café-au-lait spots and left kidney abnormality. He was confirmed as being affected by FA due to high levels of DEB-induced chromosomal breakage in metaphases of peripheral blood lymphocytes at birth (40). We identified the patient's primary fibroblast cells as being defective in *UBE2T* by overexpression of the wildtype *UBE2T* cDNA as a candidate FA gene (RefSeq: NM_014176.3) which entirely corrected G2/M phase arrest and also other cellular phenotypes induced by MMC. Importantly, no mutation in the *UBE2T* locus could be detected in the patient's germ-line DNA by Sanger sequencing or next-generation sequencing of *UBE2T*, as he had inherited genomic rearrangements at the two identical 311-bp AluYa5 elements present in the same orientation in introns 1 and 6 of the human *UBE2T* gene.

**Figure 1.**
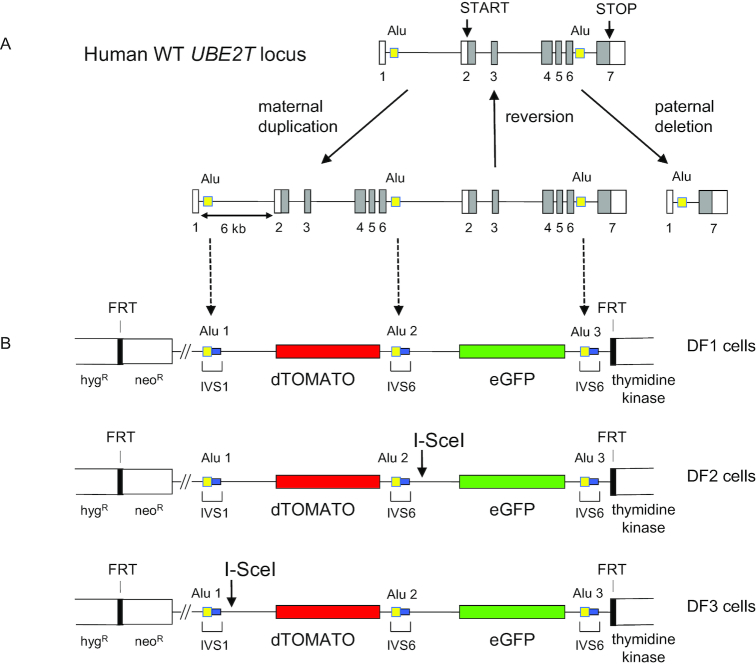
Modeling of the expanded UBE2T locus in dual fluorescence (DF) cells. (**A**) Wild type UBE2T gene, and maternal and paternal genotypes. Exons are numbered 1–7; yellow boxes, AluYa5 sequences; unfilled boxes, noncoding exons; filled boxes, coding exons. (**B**) DF1 cells contain a single genomic integrant at the FLP recombinase target (FRT) site in HeLa/406 cells, containing three identical AluYa5 repeats (yellow) and portions of intervening sequences IVS 1 and IVS 6 flanking the Alu repeats (blue boxes). The first and second Alu repeats are separated by a dTomato fluorescent protein gene (red) driven by the hPGK promoter; the second and third Alu repeats are separated by an eGFP gene (green) driven by the SFFV U3 promoter. In the DF2 cell line, an I-*Sce1* cleavage site separates the dTomato and eGFP marker genes. In the DF3 cell line, an I-*Sce1* cleavage site is located upstream of the dTomato marker gene. Thin lines, vector sequences. hyg^R^ (hygromycin resistance), neo^R^ (G418 resistance) and TK (HSV thymidine kinase, ganciclovir sensitivity) are selection markers for integrant construction (Methods).

Notably, three Alu-mediated recombination events were evident at the *UBE2T* locus In the *FANCT^−/−^* 100166/1 proband (40). From his heterozygous father, the patient had inherited a large genomic deletion of exons 2–6, resulting in an allele without any protein-coding transcript. From his healthy mother, the patient inherited a *UBE2T* allele in which a duplication of exons 2–6 had occurred, resulting in a *UBE2T* locus with three identical AluYa5 repeats. Importantly, this maternal allele was capable of expressing a transcript for a truncated UBE2T protein that contained the complete ubiquitin binding (UB) domain of UBE2T (40). When overexpressed, this shorter protein completely restored the defects in the FA pathway in *UBE2T^−/−^* cells (40). However, western blot analysis revealed that no mutant UBE2T protein was expressed from the duplicated maternal allele in either the patient's or his mother's cells, as the mRNA from this allele was subject to nonsense mediated RNA decay (40). The third recombination event in the *UBE2T* locus occurred somatically *in utero* in a hematopoietic stem cell, as the patient's peripheral blood lymphocytes were already a mixture of normal and FA-deficient cells when analyzed by chromosomal breakage three days after birth (40). Here, it is safe to hypothesize that the normal *UBE2T* allele was generated by intrachromosomal SSA or unequal sister chromatid homologous recombination between the maternally duplicated Alu elements (Figure 1A), as no normal allele that could serve as a recombination donor is present in the patient's cells. Sequencing of *FANCT^−/−^* 100166/1 proband genomic DNA PCR products corroborated that the reversion had occurred at the AluYa5 repeats within the UBE2T locus (40). Subsequently, this ‘corrected’ hematopoietic stem cell repopulated the entire hematopoietic system with normal progeny - a phenomenon known as somatic mosaicism in FA (41)—and the patient had normal peripheral blood counts for more than 15 years and never experienced bone marrow failure.

The two main branches of homology directed recombination (HDR) are RAD51-independent single strand annealing (SSA) (42) and RAD51-dependent homologous recombination (HR) (43). To develop a model to emulate Alu-mediated homology directed recombination events in the *UBE2T* locus and also for other loci in the genome, we generated dual fluorescent reporter constructs using two independent expression cassettes for green (eGFP) and red (dTomato) fluorescent proteins with three identical AluYa5 repeats in the same orientation. An exogenous I*-Sce1* site was included at either of two distinct locations in the reporter constructs to allow introduction of a single site-specific DNA double strand break (DSB). After stable integration of one copy of the dual fluorescent reporter construct into the genome of HeLa cells, we show that expression of the I*-Sce1* protein in the cells promotes DNA breakage and homology-directed AMR that mimics the reversion which had happened in the patient's hematopoietic cells.

Using the dual fluorescence system, we find that UBE2T has a limited role in HR, and demonstrate the role of HDR in Alu-mediated recombination in *UBE2T* using inhibitors or knockdown of HDR or nonhomologous end joining (NHEJ)-related proteins. Our results show that the dual fluorescent HeLa cells are also a robust tool for the systematic study of Alu-mediated recombination events and their role in inducing human disease. Combined with knockdowns of specific genes of interest, the dual fluorescence system can quantitatively report on the contribution of specific proteins to HDR and NHEJ.

## MATERIALS AND METHODS

### Dual Fluorescence (DF) plasmid constructs

The plasmids used in this work were constructed to avoid regions of homology with the resident vector at the ectopic integration site (44) or within the dual fluorescence vectors other than the Alu/IVS elements. Standard cloning methods were used to construct the vector integrated in DF3 cells from the following components: *LacZ* (nt 1–120, 6917–7374); AluYa5 (nt 135 – 445); *UBE2T* intron 1 (nt 446–626); I-*SceI* recognition (nt 632–662); *hPGK* promoter (nt 663–1204); dTomato (red fluorescent protein, nt 1222–1927); *bGHpA* (nt 1936–2152); AluYa5 (nt 2167–2478); *UBE2T* intron 6 (nt 2478–2658); SFFV U3 promoter (nt 2696–3036); eGFP (enhanced green fluorescent protein, nt 3072–3792); ΔU3 (HIV 3′ LTR partial sequence, nt 3885–3939); R (HIV-1 partial sequence, nt 3940–4034); U5 (HIV-1 partial sequence, nt 4035–4120; AluYa5 (nt 4156–4467); *UBE2T* intron 6 (nt 4467–4647; FRT site (nt 4724–4772); neomycin phosphotransferase gene (nt 4781–5576); SV40 polyadenylation sequences (nt 5577–6917); chloramphenicol acetyltransferase gene (nt 7822–8482); pSC101 origin of replication and RepA binding site (nt 10298–9406). Plasmids used to construct other cell lines were derived from the same components. Further details are available from the authors. As shown in Supplementary Figure S1 and confirmed by the present results, there is insufficient homology between the red and green fluorescent protein genes to enable homologous recombination.

### Cell culture

DF cell lines were constructed by FLP recombinase mediated integration into HeLa/406 cells and drug selection as described previously (45–50). All DF cell lines were cultured in Dulbecco's modified Eagle medium (DMEM, Gibco) supplemented with 10% newborn calf serum (NCS) at 37°C, 5% CO_2_. The human osteosarcoma (U2OS, ATCC HTB-96) DR-GFP cell lines were grown in DMEM supplemented with 10% fetal bovine serum (FBS) at 37°C, 5% CO_2_.

### Expression of I-*Sce1* endonuclease

Stable DF cell lines plated at 6 × 10^5^ cells were trypsinized and transfected with 8 ug I*-Sce1* plasmid and 10 μl of Lipofectamine 2000 (Invitrogen) per manufacturer's protocol in a six-well plate (Falcon). Medium was replaced after 24 h to remove transfection complexes. The I-*Sce1* plasmid produces an HA-tagged form of the I*-Sce1* endonuclease. Peak expression of I-*Sce1* endonuclease was at 24 h and was undetectable after 72 h by western blot (Supplementary Figure S2). Cells were grown for 8 days after I-*Sce1* transfection and split accordingly until harvested for flow cytometry. The I-*Sce1* plasmid was a gift from John Turchi (Indiana University School of Medicine).

### siRNA treatment

DF2 cell lines were plated at 4 × 10^5^ cells/well in a six-well tissue culture plate (Falcon). The cells were trypsinized and transfected with 100 nM siCtIP siRNA (Hs_RBBP8 SI1027416, Qiagen) and 10 μl of Lipofectamine 2000 (Invitrogen) per manufacturer's protocol. The siRNA is a pool of an equimolar mixture of four different siRNAs targeting the same transcript. Control experiments were performed in parallel using a non-targeting AllStars siRNA (Qiagen SI03650318). At 24 h, cells were trypsinized and transfected with 8 ug I-*Sce1* plasmid and 10 μl of Lipofectamine 2000 to allow for 48 h siRNA treatment by the time I-*Sce1* had reached peak expression at 24 h.

### Small molecule inhibitor treatments

Small molecule inhibitors were used at the following final concentrations: RAD51i (B02, Sigma SML0364, 10 uM; RI-1, Sigma SML1274, 40 uM; RI-2, Sigma SML1851, 20 μM, 30 μM), DNA-PKcs (NU7026, Selleckchem S2893, 10 μM), caffeine (Sigma C0750, 2 mM), ATMi (KU60019, Sigma SML1416, 1 μM). The inhibitors were added to the cell culture medium at the time of I*-Sce1* plasmid transfection. 6 × 10^5^ cells were trypsinized and transfected with 8 ug I*-Sce1* plasmid, inhibitor and 10 μl of Lipofectamine 2000 (Invitrogen) per manufacturer's protocol in a six-well plate. Each inhibitor was used for 3 days after transfection for efficient inhibition throughout the time of I*-Sce1* expression.

### Cell sorting (FACS)

DF2 cell lines were subjected to I*-Sce1* transfection and allowed 8 days of recovery before flow cytometry to allow turnover of preexisting fluorescent proteins. The heterogeneous population (∼5 × 10^6^ cells) was then prepared for cell sorting by centrifugation at 300 × g for 3 min, 4°C. The pellet was resuspended in 1 ml FACS buffer (Hank's Balanced Salt Solution, 25 mM HEPES, 1 mM EDTA, 1% BSA and 2% FBS) and filtered through a 35 μm cell strainer tube (Falcon). Cell sorting was performed at the Cincinnati Children's Hospital Medical Center (CCHMC) Research Flow Cytometry Core (RFCC) on a BD FACS Aria II flow cytometer with two 96 well tissue culture plates (single cell per well) (Corning) for each of the four flow cytometry quadrants. Single cell clones from each well were transferred to 10 cm tissue culture dishes (Corning). Once the 10 cm dishes were ∼80% confluent the cells were harvested for DNA analysis. DNA was isolated using an EZNA tissue isolation kit (Omega Bio-Tek) to serve as template in PCR amplifications.

### Polymerase chain reaction

PCR was performed using Lac-Forward (5′-CTTCAAATCCGACCCGTAGA-3′) and TK-Reverse (5′-GTAAGTCATCGGCTCGGGTA-3′) primers. PrimeSTAR GXL polymerase (Takara) was used per manufacturer's instructions for 50 μl reactions using 120 ng template. Internal control primers (Figure 3) were: forward 5′-CCCAACCTACACTAACCTTAACC and reverse 5′-CCACACCAACCTCCTCATAAT. Cycling conditions were as follows: denaturation, 98°C, 10 s; annealing, 57°C, 15 s; extension, 68°C, 1 min for 30 cycles. PCR products were purified with an EZNA Cycle Pure kit (Omega Bio-Tek) and 20 μl of each purified product was electrophoresed on a 1% ultra-pure agarose gel (Invitrogen) to verify the sizes of the recombination products.

### Flow cytometry

Flow cytometry was performed on the BD Accuri C6 flow cytometer. 5 × 10^5^ adherent cells were trypsinized (Gibco) and centrifuged at 300 × g for 3 min. Supernatant was removed and the cell pellet was resuspended in 300 μl PBS. The cells were centrifuged for another 3 min at 300 × g. The supernatant was aspirated and the cell pellet was resuspended in 200 μl PBS. The number of events counted was set to 20 000 on medium flow. Color compensation was set at correcting FL2 by subtracting 6.3% of FL1. The color compensation and quadrant determination was determined empirically to minimize spectral overlap using marker DF6 and DF7 cell lines that produce only red fluorescent dTomato protein or green fluorescent eGFP protein respectively.

### CRISPR-Cas9 knockdown

CRISPR guides that target *UBE2T* were selected using the design website http://crispr.mit.edu (51). Guide 2 (TTTGATACCTACGAGCTCGCAGG) was chosen after single cell cloning of transduced cells and western analysis. This guide RNA is complementary to intron 1, approximately 1.3 kb downstream of the *UBE2T* 5′ Alu element. Briefly, LentiCRISPR v2 containing the puromycin resistance-mediating *pac* gene was a kind gift from Feng Zhang (Addgene plasmid #52961). Cloning of the guide RNA into the LENTICRISPR v2 vector was performed as specified by the Zhang laboratory protocol (52). Infectious replication-deficient lentiviral particles in the VSV-G pseudotype were generated as previously described (40). DF3 and U2OS DR-GFP cell lines were transduced with a lentivirus construct expressing Cas9 and the *UBE2T* CRISPR sgRNA at an MOI of <1 and single-cell clones were selected by puromycin treatment. Single cell clones were tested by western blot for loss of UBE2T expression (40). Control cell lines underwent the same procedures but were transduced with the empty LentiCRISPR v2 vector that does not produce the *UBE2T* CRISPR guide.

### Retroviral UBE2T expression vectors

In order to demonstrate that UBE2T deficiency of *UBE2T^−/−^* HeLa cells generated through CRISPR/Cas9 was indeed responsible for the DNA repair defects, a retroviral vector was constructed that overexpressed the wild-type *UBE2T* cDNA. As the *UBE2T^−/−^* HeLa cells were already resistant to neomycin, puromycin and hygromycin, the cDNA of the blasticidin resistence gene was cloned from the pHAGE.CMV.EG_SLX4 vector (a kind gift of Agata Smogorzewska, Rockfeller University, NY, USA) and inserted into the retroviral pS91 UBE2T-IRES-puroR vector using *Nco*I-*Cla*I. The final retroviral vector expressed the blasticidin resistence gene after the IRES site. Infectious recombinant retroviral particles with the VSV-V phenotype were produced in 293T cells and used to transduce the HeLa cells at a multiplicity of infection of <0.2, as described previously (40).

## RESULTS

### Construction of dual fluorescence (DF) reporter constructs for the maternal *UBE2T* locus

The wild-type *UBE2T* allele in humans comprises seven exons with the translation start in exon 2, and two identical AluYa5 elements in the same orientation (www.ensembl.org). The first Alu element is located ∼180 bp 3′ of the exon1/intron 1 border, and a second Alu element is ∼180 bp 3′ of the exon 6/intron 6 border (Figure 1A). The mutant maternal allele contains a duplication of exons 2–6 and the mutant paternal allele exhibits a deletion of exons 2–6. Both mutant alleles seem to have occurred by Alu-mediated recombination. We have shown previously that the paternal allele is present as a founder mutation at low frequencies in the Italian and the German populations. However, we did not detect the maternal duplication in almost 2000 individuals (40) suggesting that it is much more restricted to the maternal lineage. In the proband, spontaneous deletion of the maternally duplicated exons 2–6 by recombination between neighboring Alu elements has been postulated to account for the outgrowth of normal blood cells and the reversion of the hematopoietic phenotypes (40).

To simulate Alu-mediated recombination in the *UBE2T* locus, we generated clonal cell lines in which one copy of a model *UBE2T* locus was integrated into the single FLP recombinase target (FRT) site in HeLa/406 cells (45–49,53) (Figure 1B). Previous work has shown that Alu-mediated recombination is responsible for homozygous deletions in the *STK11/LKB1* locus of HeLa cells to generate aberrant LKB1 fusion transcripts (54), for *MLL/KMT2A* duplications in normal and AML hematopoietic cells (55), and for nonallelic homologous recombination deletions in the FANCA, B, C and D2 genes (5,56) in FA patient cells.

To model the maternal *UBE2T* allele containing the duplication, three AluYa5 repeats and ∼180 bp of *UBE2T* flanking intron 1 (IVS1) or intron 6 (IVS6) sequences were separated by complete reporter gene cassettes encoding the red fluorescent protein (RFP) dTomato driven by the human phosphoglycerate kinase (PGK) promoter with a 3′ bGH polyA site, and the eGFP protein driven by a modified viral SFFV U3 promoter region followed by the polyA sequences of a SIN lentiviral LTR (57).

There is no sequence overlap between the promotors and polyA sites of the expression cassettes. In addition, the dTomato and eGFP ORFs have minimal sequence overlap and only two identical nucleotide stretches at the 5′ and 3′ ends of 21 and 25 bp (Supplementary Figure S1), respectively. For clarity, we have numbered the AluYa5 elements Alu 1, 2 and 3 in our reporter constructs (Figure 1B), although these elements comprise the identical 311 bp sequence. Dual Fluorescence 1 (DF1) cells do not contain a cleavage site for I*-Sce1*, while in Dual Fluorescence 2 (DF2) cells a cut site for I*-Sce1* preceded the eGFP gene (Figure 1B). In DF3 cells the I-*Sce1* cut site was placed upstream of the dTomato gene (Figure 1B).

### Dual fluorescence cells to monitor the repair of DSBs in the presence of Alu repeats

**Figure 2.**
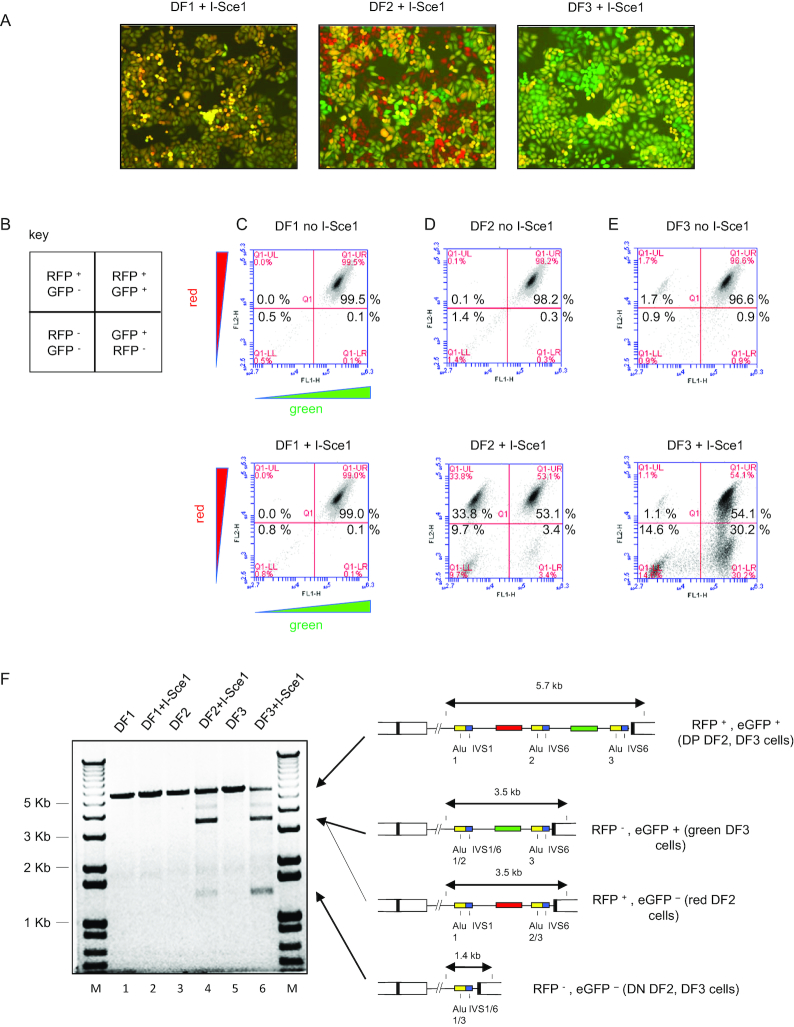
DSB-induced recombination in DF cells. (**A**) I-*Sce1* expression does not cause loss of either color marker gene in DF1 cells, but leads to preferential loss of eGFP expression in DF2 cells, and preferential loss of dTomato expression in DF3 cells. (**B**) Key to flow cytometry profiles. In the absence of I-*Sce1* digestion cells contain dTomato (RFP+) and eGFP (GFP+) marker genes and appear in the upper right quadrant (DP, double positive). Loss of the eGFP gene renders cells red (R, upper left, RFP+, GFP–). Loss of the dTomato gene renders cells green (G, lower right, GFP+, RFP–). Loss of both marker genes renders cells double negative (DN, lower left, RFP–, GFP). (**C**–**E**) flow cytometry profiles of untreated (no I-*Sce1*) or I-*Sce1* transfected (+ I-*Sce1*) (Methods) DF1, DF2, or DF3 cells, respectively. The data shown are representative of four or more experiments on each cell type. F, PCR analysis of control and I-*Sce1* treated DF cell lines. •, I-*Sce1*-dependent aberrant recombination product.

DF1, 2 and 3 cells initially expressed both red dTomato and green eGFP markers, and appeared yellow under UV illumination (Figure 2A). These cells were then transfected with a plasmid that expressed the I*-Sce1* endonuclease maximally at 24 h post transfection (Supplementary Figure S2) to introduce a unique DSB within the DF2 and DF3 reporter constructs. As shown in Figure 2A, DF1 cells did not change color (yellow) following transfection with the I*-Sce1* expression plasmid as the DF1 construct does not harbor an I*-Sce1* site. In contrast, a substantial fraction of DF2 cells showed red fluorescence associated with the loss of eGFP (RFP+, eGFP–) and only a smaller proportion of cells turned green (RFP– eGFP+) after I*-Sce1* transfection, while DF3 cells preferentially turned green (RFP–, eGFP+). These data suggest that a single DNA double strand break between identical Alu repeats can efficiently induce recombination events leading to loss of the intervening color reporter gene, consistent with Alu-mediated intrachromosomal SSA or unequal homology-dependent recombination between sister chromatids.

### Quantitation of homology directed recombination events by flow cytometry

DF1, 2 and 3 cells were initially >95% double positive (DP) (RFP+, eGFP+) (Figure 2B–E). It is predicted that homologous recombination between proximal Alu elements would produce red DF2 cells by Alu 2/3 recombination and green DF3 cells by Alu 1/2 recombination, while recombination between distal Alu 1/3 elements would produce double negative cells. We observed that *in vivo* I*-Sce1* expression did not significantly change the flow profile of DF1 cells which lack an I*-Sce1* site (Figure 2C). However, representative results show that after I*-Sce1* transfection, >30% of DF2 cells lost the eGFP marker (red, RFP+, eGFP–; upper left quadrant) (Figure 2D), and >30% of DF3 cells lost the dTomato marker (green, RFP–, eGFP+; lower right quadrant) (Figure 2E). Approximately 10% of DF2 or DF3 cells lost both color markers (double negative (DN) RFP–, eGFP–; lower left quadrant), most probably due to recombination between the outermost Alu elements (Alu 1/3).

To confirm the extent of I*-Sce1* induced deletions, we performed PCR across the ectopic integration site in each of the reporter cell lines. Lanes 1 and 2 (DF1 and DF1+I*-Sce1*) showed no deletions in the reporter construct in the DF1 cells upon I*-Sce1* expression (Figure 2F), as anticipated. In contrast, I*-Sce1* digested DF2 and DF3 cell DNAs displayed distinct lower molecular weight major bands (Figure 2F, lanes 4, 6) corresponding to deletion products expected of recombination between the proximal (Alu 1/2) and distal (Alu 2/3) Alu sites, consistent with the changed flow cytometry patterns of these cells after I*-Sce1* expression. Specifically, recombination between the homologous Alu 1 and Alu 2 sites was found to generate a major PCR band of approximately 3.5 kb after deletion of the dTomato gene in RFP–, eGFP+ (green) DF2 cells (3.4% of total cells) and DF3 cells (30.2% of total cells).

Recombination between Alu 2 and Alu 3 generated a major PCR band of approximately 3.5 kb and deleted the eGFP gene in RFP+, eGFP– (red) DF2 cells (33.8% of total cells) and DF3 cells (1.1% of total cells). Recombination between Alu 1 and Alu 3 generated a major 1.4 kb PCR band and deleted both the dTomato and eGFP genes in RFP–, eGFP– (double negative) DF2 (9.7% of total cells) or DF3 cells (14.6% of total cells). Thus, in I-*Sce1* treated DF2 or DF3 cells, more than ∼45% of the cells underwent recombination (red, green, double negative cells).

As described in detail below, we estimated the percentage of DF2 double positive cells that had undergone recombination *in vivo* after I*-Sce1* transfection, by *in vitro* I*-Sce1* digestion of a full-length PCR product spanning the DF2 ectopic site. Since the DF2 full-length PCR product could only have come from double positive cells, and more than ∼60% of this PCR product was resistant to *in vitro* I*-Sce1* cleavage (Figure 4I), these results indicate that >75% of the ectopic sites (45% recombinant cells plus 60% of double positive cells (53.1% of total) had been cut by I*-Sce1 in vivo* and then further processed by DNA repair. Thus, Alu-mediated homology-directed recombination events had occurred in >30% of DF2 cells (red) and DF3 cells (green) following introduction of the I*-Sce1* DSB within the reporter constructs. However, based on the resistance of the ectopic site PCR product to *in vitro* I*-Sce1* digestion, additional repair mechanisms (e.g. NHEJ/MMEJ) were also active in >60% of cells where both fluorescent reporters were retained.

### Single colony PCR characterization of recombinants

**Figure 3.**
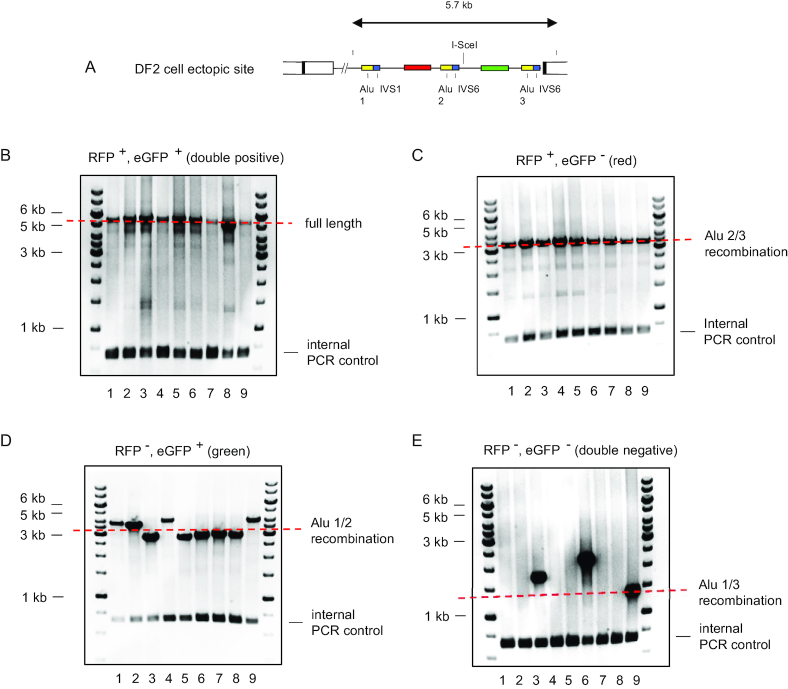
PCR analysis of flow sorted DF2-derived cell clones detects homology directed and nonclassical recombination. DF2 cells were treated with I-*Sce1*. Eight days later, DNA was isolated from each of thirty-six flow sorted colonies and analyzed by PCR. (**A**) map of the DF2 cell ectopic site construct; (**B**) RFP+, GFP+ cell clones; (**C**) RFP+, GFP– cell clones; (**D**) RFP–, eGFP+ cell clones; (**E**) RFP–, eGFP– cell clones.

Because standard PCR on DNA from unsorted cultures may not disclose recombination products that arise from minor percentages of the total cell population, we took a single colony PCR approach to assess the fluctuation between cells following I*-Sce1* digestion. Eight days after I*-Sce1* plasmid transfection, DF2 cells were FACS sorted into individual double positive, red, green, and double negative cells and clonally expanded. Genomic DNA was harvested from randomly selected clones and amplified by PCR with primers across the ectopic site. In DNA from double positive (RFP+, eGFP+) DF2 cells, PCR across the single ectopic site (Figure 3A) generated a major band of ca. 5.7 kb in addition to bands of lesser intensity that presumably resulted from recombination during clonal expansion (Figure 3B). The majority of red (RFP+, eGFP–) cells displayed a major band of ca. 3.3 kb, consistent with recombination by sister chromatid HR or intrachromosomal SSA between the Alu 2 and Alu 3 elements flanking the I*-Sce1* site (Figure 3C).

None of the colonies that turned green (RFP–, eGFP+) showed the ca. 3.3 kb band predicted by homology-directed repair between Alu 1 and Alu 2, but apparently had undergone a more complex series of recombinations generating smaller and larger PCR products that retained the eGFP gene and PCR primer binding sites (Figure 3D). Surprisingly, only a minority of the double negative (RFP–, eGFP–) cell clones showed the expected recombination between Alu 1 and Alu 3 (Figure 3E, lane 9).

The differences in the PCR profiles of the DNA from unsorted vs. sorted cells was striking. Following PCR of DNA from unsorted cells (Figure 2F), the most abundant cell population gave the expected ca. 3.5 kb PCR product, although these may still have comprised a minor percentage of the total cells. In contrast, in the PCR of DNA from sorted and cloned cells (Figure 3), even the lesser abundance green cells could give products that were different from the expected Alu 1/2 recombinant.

The data of Figures 2 and 3 argue that the most plausible mechanism of *in vivo* reversion of the maternally duplicated *UBE2T* gene resulted from a spontaneous double strand break that led to homology-directed recombination between Alu 2 and Alu 3 elements. Additionally, it is likely that a substantial percentage of breaks did not recombine by classical RAD51-dependent homologous recombination to restore the WT allele, but instead resulted in cells carrying alternative recombinations that were selected against in the patient's hematopoietic compartment.

### Role of DNA repair proteins in Alu-mediated recombination at the model *UBE2T* locus

**Figure 4.**
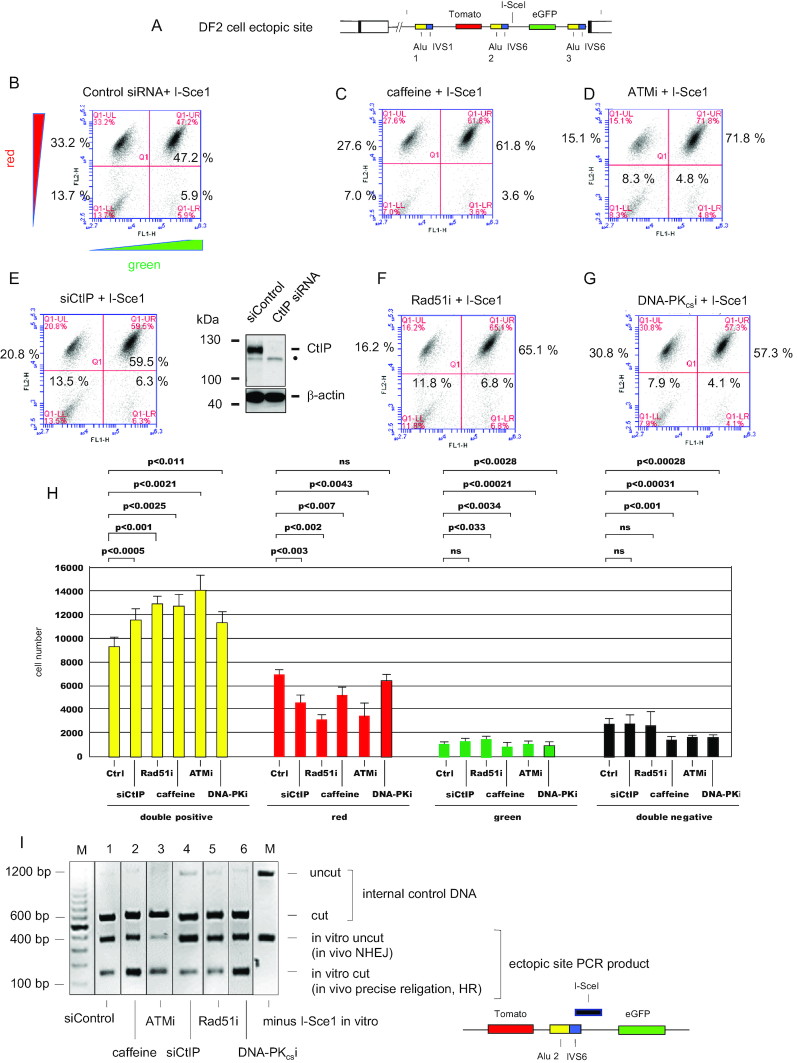
Recombination in DF cells involves canonical HR and noncanonical pathways. (**A**) map of the DF2 cell ectopic site construct. (B–G) Representative flow cytometric analyses of DF2 cells treated with I-*Sce1*. (**B**) Control siRNA; (**C**) caffeine; (**D**) ATM inhibitor KU60019; (**E**) left panel, siCtIP siRNA #1 + #2, right panel CtIP knockdown (western blot), •, crossreacting band; (**F**) RAD51 inhibitor BO2; (**G**) DNA-PKcs inhibitor NU7026. (**H**) Quantitation of flow cytometry results. Data are the means + S.D. of three biological replicates each assayed in triplicate (paired Student's *t*-test). Values of *P* > 0.05 were considered not significant (ns). I, *In vitro* I-*Sce1* digestion of ectopic site PCR products from I-*Sce1* transfected DF2 cells (B–G, see text for details). An enlargment of part of the DF2 cell ectopic site construct is shown to illustrate the PCR product (black bar) used for *in vitro* I-*Sce1* digestion. Borders indicate that samples are from nonadjacent gel lanes.

To test the mechanism of recombination in the model *UBE2T* locus further, DF2 cells were exposed to I*-Sce1* while proteins involved in nonhomologous end joining (NHEJ) or HR were chemically inhibited or knocked down by siRNA. First, we treated I*-Sce1* transfected DF2 cells with caffeine (Figure 4A), which preferentially inhibits the major DNA repair kinases ATM (ataxia telangiectasia mutated) and ATR (ataxia telangiectasia and RAD3-related) (58–60). Compared to control cells digested with I*-Sce1* (Figure 4B), caffeine significantly decreased the percentage of red, green, and double negative cells, and consequently increased the fraction of double positive cells (Figure 4C, H). This effect on recombination efficiency was more dramatic when using the ATM specific inhibitor KU60019 (Figure 4D, H), consistent with the stimulation of DSB end resection and downstream steps in HR and NHEJ by ATM (58–62).

The CtIP (C-terminal binding protein 1 interacting protein) nuclease catalyzes an early step in end resection of DNA DSBs in conjunction with the MRN complex, thereby terminating NHEJ and initiating HR (63–66). siRNA-mediated inhibition of CtIP (Figure 4E, H) or chemical inhibition of the RAD51 recombinase (RAD51i B02, Figure 4F, H; RAD51i RI-1, RAD51i RI-2, Supplementary Figure S3) also significantly decreased the homology-directed recombination that produced red cells upon I*-Sce1* digestion. In contrast, specific inhibition of the NHEJ protein DNA-PK_cs_ by NU7026 (67) did not significantly change the percentage of red cells (Figure 4G, H), as expected if RFP+, eGFP– cells resulted from HR or SSA. Inhibition of NHEJ by NU7026 decreased the percentage of double negative and green cells, but increased the percentage of double positive (yellow) cells, most likely by promoting precise religation of the I*-Sce1* cut (68).

To distinguish between *in vivo* precise religation vs. mutagenic NHEJ of the cleaved I*-Sce1* site, we carried out PCR across the ectopic site in DNA from DF2 cells transfected with I*-Sce1*. We then digested the PCR product with I*-Sce1 in vitro* (Figure 4I). DF2 cells transfected with I*-Sce1* and treated with caffeine or ATMi showed increased *in vitro* cutting of the ectopic site PCR product relative to cells treated with siControl (Figure 4I, lanes 1–3). Inasmuch as these cells displayed reduced homology-directed repair (i.e. red cells, Figure 4C, D, H), we conclude that the increased *in vitro* cutting of the PCR product reflected an increase in precise ligation in vivo. In contrast, the ratio of *in vitro* cut to uncut PCR product was reduced in DNA from cells treated with siCtIP or RAD51i (Figure 4E, F, H). Finally, inhibition of NHEJ by DNA-PK_cs_i resulted in an increase in the ratio of cut vs. uncut PCR product (Figure 4I, lane 6). Since DNA-PK_cs_i did not significantly change HDR (Figure 4H), we presume this increased cutting *in vitro* is due to enhanced precise ligation when DNA-PK_cs_ and NHEJ end processing are inhibited *in vivo*.

Based on the results of PCR in the FACS sorted cell clones (Figure 3D, E), we hypothesize that NHEJ was involved in the aberrant recombination occurring in the double negative (RFP–, eGFP–) and green (RFP–, eGFP+) I*-Sce1* transfected DF2 cells. While the results of CtIP knockdown and RAD51i treatment are consistent with a reduction in HR, CtIP is also required for SSA (69), and knockdown of CtIP end resection can also be compensated by increased NHEJ (70,71), which would reduce *in vitro* I*-Sce1* cutting. Although small-molecule RAD51 inhibitors have been shown to reduce HR (72–75), the possibility of off-target effects of these compounds remains.

**Figure 5.**
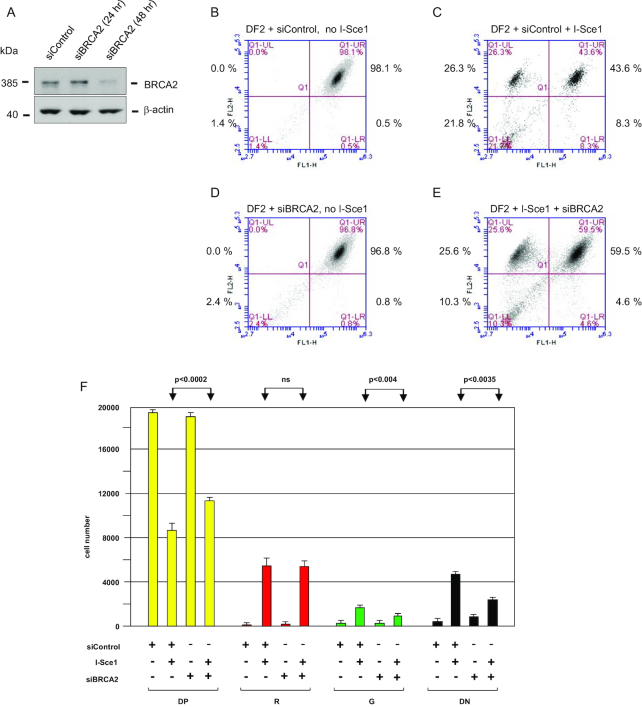
BRCA2 knockdown does not affect Alu-mediated SSA, but enhances end joining and mutagenic recombination. (**A**) Western blot of whole cell extracts of DF2 cells treated with siControl or siBRCA2. (**B**) Flow cytometry profile of DF2 cells treated with control siRNA. (**C**) Flow cytometry profile of DF2 cells treated with control siRNA and I-*Sce1*. (**D**) Flow cytometry profile of DF2 cells treated with BRCA2 siRNA. (**E**) Flow cytometry profile of DF2 cells treated with BRCA2 siRNA and I-*Sce1*. (**F**) Quantitation of flow cytometry results. Values are means + S.D. of three biological replicates each assayed in triplicate (paired Student's *t*-test). *P* values compare I-*Sce1* treated samples ± BRCA2 siRNA.

Therefore, we sought an additional test of whether RAD51-dependent HR was responsible for contraction of the model UBE2T locus that produced red (RFP+, eGFP–) DF2 cells. As shown in Figure 5, we assayed the effects of knocking down BRCA2 (Figure 5A), which interacts with RAD51 and controls its translocation into the nucleus, nucleofilament formation and assembly of RAD51 foci in response to DNA damage (76–79). I-*Sce1* was expressed in DF2 control cells or cells treated with *BRCA2* siRNA (Figure 5B-F). The percentage of double positive (RFP+, eGFP+) cells increased significantly with BRCA2 knockdown (Figure 5F). By contrast, BRCA2 knockdown had no effect on the fraction of red cells produced by I*-Sce1* expression. Instead, the percentage of green (RFP–, eGFP+) and double negative (RFP–, eGFP–) cells both decreased when BRCA2 was depleted. Conversely, the fraction of double positive cells was increased by compensating NHEJ/MMEJ, and mutagenic recombinations which produce green and double negative cells (Figure 3) were also enhanced. Thus, t*he* change in the percentages of double positive, double negative and green cells indicates the biological effectiveness of the BRCA2 knockdown, while the absence of an effect on the percentage of red cells argues that BRCA2-independent SSA is responsible for the DF2 Alu-mediated contraction, and that downregulation of HR by BRCA2 depletion promotes alternative, mutagenic forms of recombination (80–83).

### *Cis*-acting effects of the Alu 1/IVS1 sequence on recombination at the model UBE2T locus

**Figure 6.**
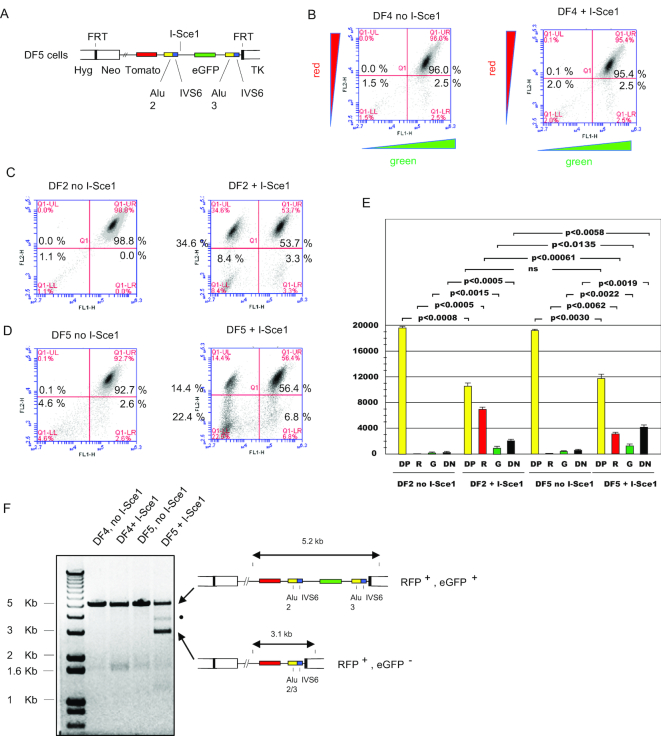
*Cis*-acting effects of the Alu 1/IVS1 sequence on recombination at the model UBE2T locus. (**A**) DF4 cells contain the same genomic integrant as DF1 cells except for deletion of the first AluYa5 element and IVS1 fragment. DF5 cells contain the same integrant as DF4 cells, but also contain an I-*Sce1* site upstream of the eGFP gene (as in DF2 cells). (**B**–**D**) flow cytometry profile of DF4, DF2 or DF5 cells, respectively, either untreated or transfected with I-*Sce1*. (**E**) Quantitation of flow cytometry results. DP, double positive; R, red; G, green; DN, double negative. Data are the means + S.D. of three biological replicates each assayed in triplicate (paired Student's *t*-test). Values of *P* > 0.05 were considered not significant (ns). F, PCR analysis of control and I-*Sce1* treated DF4 and DF5 cell lines. •, I-*Sce1*-dependent aberrant recombination product.

Digestion of DF2 cells with I*-Sce1* resulted in the expected 1.4 kb Alu 1/3 recombination product when DNA from unsorted cells was analyzed (Figure 2F), however, only about 9% of the cells fell in the double negative population (Figure 2D) and only 1 of 9 randomly selected double negative cell clones produced the 1.4 kb PCR product predicted to arise from Alu1/3 recombination (Figure 3D). To determine if Alu 1/IVS1 was exerting an effect in *cis* on homology directed recombination between Alu2/3, two additional cell lines, DF4 and DF5, were constructed that deleted Alu 1/IVS1 (Figure 6A). The DF4 cell line differs from DF5 in that DF4 does not contain an I*-Sce1* site; consequently, I*-Sce1* transfection did not change the flow cytometry profile of DF4 cells (Figure 6B). I*-Sce1* digestion of DF2 or DF5 cells resulted in similar percentages of double positive cells (Figure 6C, D). Surprisingly, I*-Sce1* expression in DF5 cells (Figure 6C) produced approximately half as many red (RFP+, eGFP–) cells and twice as many double negative (RFP–, eGFP–) recombinants as in I*-Sce1* digested DF2 cells (Figure 6D). This result indicates that Alu 1/IVS1 acts in *cis* to promote Alu 2/3 homology-directed recombination to yield red cells, and to suppress recombination leading to double negative cells.

The decrease in the percentage of DF5 red cells after I*-Sce1* transfection is not due to reduced efficiency of I*-Sce1* digestion, as the total percentage of red, green and double negative cells was closely similar between I*-Sce1* digested DF2 and DF5 cells, but the ratio of green:red:double negative recombined cells changed from approximately 1:10:2.5 in DF2 cells to approximately 1:2:3 in DF5 cells. To analyze the I*-Sce1* induced deletions, we performed PCR across the ectopic site in DF4 and DF5 cells. The gel of Figure 6F confirmed that I*-Sce1* digested DF5 cells displayed a lower molecular weight band of ∼3.1 kb corresponding to the deletion product expected for recombination at the Alu 2 and Alu 3 sites, consistent with the flow cytometry patterns of these cells (Figure 6C, D; RFP+, eGFP–).

We also observed that ∼22% of the I*-Sce1* digested DF5 cells were double negative (RFP–, eGFP–). Taken with the PCR results on double negative cell DNA in Figure 3C, we judged that amplified PCR products smaller than 3.1 kb retained the PCR primer binding sites and were the result of excessive nuclease digestion at the I*-Sce1* break or spontaneous breakage and recombination at sites other than the Alu 2 and Alu 3 repeats.

### Effect of UBE2T knockout on homology directed recombination

**Figure 7.**
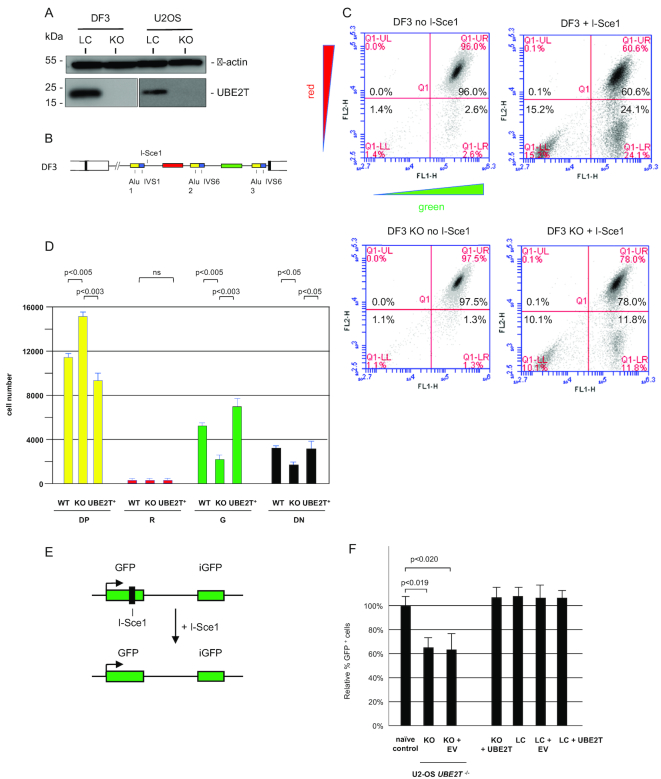
*UBE2T* knockout partially inhibits HR. (**A**) CRISPR-Cas9 knockout of *UBE2T* in DF3 and U2OS cells (western blot). Note the greater abundance of UBE2T in tetraploid DF3 cells. (**B**) Schematic of the DF3 cell line construct. (**C**) Representative flow cytometry profiles of DF3 control cells and *UBE2T* CRISPR knockout DF3 cells with or without I-*Sce1* digestion. (**D**) Quantitation of flow cytometry profiles of triplicate biological replicates assayed in triplicate. Values are the means + S.D. (paired Student's *t*-test). KO, CRISPR knockout DF3 cells; *UBE2T*^+^, KO cells reconstituted with lentiviral *UBE2T* cDNA. (**E**) DR-GFP schematic. (**F**) Quantitation of homology directed repair in U2OS control cells and *UBE2T*^−/−^ CRISPR knockout (KO) U2OS cells. Naïve, unedited U2OS cells; KO, CRISPR knockout of *UBE2T*; KO + EV, KO cells treated with lentivirus empty vector; KO + *UBE2T*, KO cells reconstituted with lentiviral *UBE2T* cDNA; LC, cells treated with control lentiviral vector. Values are means + S.D. of three biological replicates each assayed in triplicate (paired Student's *t*-test).

The conclusion that a recombination event with generation of a wild-type *UBE2T* allele in the FANCT patient is the direct consequence of homology directed recombination (HDR) and the reason for the phenotypic reversion in a hematopoietic cell containing the inherited partial duplication of the *UBE2T* gene, is based on the supposition that *FANCT/UBE2T* deficient cells can still perform HDR. Because several FA proteins, including UBE2T, have been implicated in multiple forms of DNA repair including homology directed recombination (26,84,85), we wished to test directly the effect of *UBE2T* loss on HDR efficiency. Thus, lentivirus-mediated CRISPR-Cas9 was used to knockout the *UBE2T* genes in DF3 cells (*UBE2T^Δ^* cells) (Figure 7A) and then single cell clones were expanded. Homology directed repair of the I*-Sce1* cut in DF3 cells (Figure 7B) is predicted to yield green cells (RFP–, GFP +; Figure 7C). To assess the efficiency of homologous recombination we compared the levels of green cells produced when naïve DF3 cells or *UBE2T^Δ^* DF3 cells were transiently transfected with the I*-Sce1* vector. Compared to naïve cells, *UBE2T^Δ^* cells showed an approximate 50% decrease in the percentage of RFP–, eGFP+ (green) cells (Figure 7C, D). We conclude that significant homology-directed recombination could still occur at the Alu repeats in the model *UBE2T* allele of *UBE2T^Δ^* cells when a DSB breaks occurred between flanking Alu repeats.

For comparison, the *UBE2T* genes were also knocked out in U2OS cells using CRISPR-Cas9 lentivirus (Figure 7A). The *UBE2T*^−/−^ knock-out U2OS cells contain the direct repeat (DR-GFP) construct (Figure 7E), which has been used widely as a reporter for HR (86–91). In these cells, the upstream *SceGFP* gene is inactive due to the presence of a stop codon within the I-*Sce1* cleavage site, while a second partial GFP fragment of 812 bp (*iGFP*) is present 3.7 kb downstream on the same chromosome. I-*Sce1* cleavage of this construct can lead to a homology-dependent gene conversion event between the *iGFP* and the broken *SceGFP* sequence, and thereby restore a functional GFP protein which can be measured by flow cytometry.

Biallelic CRISPR-Cas9 knockout of the U2OS cell *UBE2T* genes (*UBE2T^−/−^*) resulted in a significant decrease in GFP + cells after I*-Sce1* cleavage compared to naïve control U2OS cells or the same U2OS *UBE2T*^−/−^ cells corrected with a viral *UBE2T* cDNA expression vector (KO + *UBE2T*; Figure 7F). These results showed that the UBE2T protein contributes to the efficiency of homology dependent repair, however, it is not absolutely required for HR repair to occur. The observation that *UBE2T^−/−^* cells retained more than half of the HR activity of *UBE2T^+/+^* cells implies that redundant pathways to homology directed repair are active in these engineered FA-T cells.

## DISCUSSION

The E2 ubiquitin conjugases UBE2T and UBE2W can act in the ubiquitination of FANCI/D2. However, UBE2T is only the conjugase required for activation of the FA pathway in response to MMC/ICL, and biallelic loss-of-function mutations of *UBE2T* lead to the FA phenotype of chromosome instability (36–39,92–94). Our experiments to model recombination at the *UBE2T* locus were prompted by an FA patient with inactivating duplication and deletion mutations in *UBE2T* and whose fibroblasts were hypersensitive to the genotoxins DEB and cisplatin, and defective in monoubiquitination of FANCD2 in response to MMC (40). Surprisingly, the *UBE2T* duplication was almost completely absent in genomic DNA from the patient's peripheral blood, consistent with normal thrombocyte, leukocyte and platelet counts as well as normal bone marrow cellularity.

To test whether homology dependent recombination at the *UBE2T* AluYa5 elements could account for the genetic reversion in this patient, we integrated single copy model *UBE2T* alleles into HeLa/406 cells using FLP recombinase (45–50). The model *UBE2T* alleles contained red (*dTomato*) and green (*eGFP*) reporter genes separated by an I*-Sce1* cleavage site so that the recombination events after DNA double strand breakage could be monitored on protein levels by flow cytometry.

I*-Sce1* expression in the reporter cell lines DF2 and DF3 resulted in the cleavage of greater than 75% of the model *UBE2T* alleles and changes in color that were monitored by fluorescence microscopy and flow cytometry. The loss of the dTomato or eGFP ORFs and the sizes of the major PCR products from the I*-Sce1* treated cells were consistent with homology-dependent Alu-mediated recombination. From these results, we conclude that an endogenous DSB likely initiated recombinational reversion of the duplicated maternal *UBE2T* allele in the proband and possibly partial deletion of one *UBE2T* allele in the father.

The structures of the recombined reporter loci indicated that HDR had occurred primarily between pairs of Alu elements. However, analysis of flow sorted clonal cell populations indicated that more complex mechanisms leading to unexpected patterns of loss of both color reporter genes were also active in a minority (<15%) of cells. Knockdown or inhibition of proteins involved in DNA damage signaling and HR (RAD51, ATM, CtIP), but not NHEJ (DNA-PKcs), inhibited recombination between Alu elements, supporting the view that classical single strand annealing was responsible for Alu-mediated recombination. In DF2 cells, inhibition shifted approximately half of the recombinations from HDR (RFP+, eGFP–) to NHEJ to yield RFP+ eGFP+ cells that generated I*-Sce1* resistant PCR products.

Single strand annealing is a RAD51-independent mechanism of recombination (42,80,81,95). To test for the role of RAD51 in I*-Sce1* repair in the model *UBE2T* locus, we used the RAD51 inhibitors B02, RI-1 and RI-2, which have been used extensively to analyze RAD51-mediated recombination (72–75,96–115). Each of these drugs reduced Alu-mediated HDR by ∼50%; however, these drugs may have off-target effects in addition to inhibition of RAD51. Therefore, we also tested the DNA repair in conjunction with BRCA2 knockdown, which has been shown to block RAD51-dependent HR, and increase error-prone forms of recombination (81). Our results are consistent with these previous observations, in that BRCA2 knockdown increased the percentage of RFP–, GFP+ and RFP–, eGFP– cells, which result from aberrant recombination (Figure 3). However, BRCA2 knockdown did not affect the percentage of RFP+ eGFP– cells, the majority of which appear to have resulted from SSA (Figure 5). Although not all functions of RAD51 (e.g. S-phase focus formation (116) and replication fork reversal (117)) are dependent on BRCA2 (118), we conclude that RAD51-independent homology directed SSA is responsible for at least a large proportion of Alu-mediated recombination in our system.

We also observed that Alu 1/IVS1 acts in *cis* to promote Alu 2/3 homology directed recombination to yield red cells. In DF2 cells, Alu 1 is 2 kb upstream of Alu 2, which is well within the length of DNA that is resected in advance of homology directed repair (119,120). In flow cytometry-sorted cells (Figure 3), only one of nine double negative clones showed the expected Alu 1/3 recombination product. This result suggests that there are sequences in the construct that allow homeologous recombinations that retain (lanes 3, 6) or delete (lanes 1, 2, 4, 5, 7, 8) the PCR primer sites. One possible explanation for the positive influence of Alu 1/IVS1 on HR is that the Alu 1 sequence or its binding proteins (121–123) on the sister chromatid aid in positioning the Alu 2/IVS6 to anneal to the Alu3/IVS6 sequence during intrachromosomal SSA, akin to the phenomenon of transvection (124–127).

Since HR had been implicated in the reversion of the duplication in the *UBE2T^−/−^* patient's hematopoietic cells, we tested for this activity following *UBE2T* knockout in DF3 and U2OS cells. An approximate 50% decrease in RFP– eGFP+ cells in *UBE2T* knockout DF3 cells implied that redundant homology-dependent repair pathways are operable for Alu-mediated recombination. In *UBE2T^−/−^* U2OS cells, the DR-GFP assay showed a reproducible decrease of ∼40% in HR, suggesting that UBE2T plays a role in HR in addition to activation of FANCI/D2 for ICL removal, but that one or more UBE2T-independent parallel pathways also exist for residual HR in *UBE2T^−/−^* cells. An overview of the decrease in homologous recombination detected by the DR-GFP assay due to insufficiency of several FA or HR-related proteins (Supplementary Table S1) shows a wide range of effects, with knockdown of several known FA proteins showing incomplete inhibition of HR, as in the case of *UBE2T* knockout.

We propose that *UBE2T*-dependent homology directed recombination is one mechanism of Alu-mediated reversion of the model UBE2T locus and that in *UBE2T* null patient cells, a UBE2T-independent residual mechanism of HR such as SSA was responsible for contraction of the partially duplicated *UBE2T* locus. We conclude that a spontaneous DSB in the duplicated *UBE2T* locus of the FANCT patient that had occurred in an hematopoietic stem cell was sufficient for UBE2T independent, Alu-mediated recombination that restored a wild-type *UBE2T* gene and thereby provided survival advantage for that stem cell and its progeny.

Finally, we note that the dual fluorescence flow cytometric assay for recombination in DF cells is quantitatively responsive to DNA double strand breaks and the manipulation of DNA repair pathways, indicating that this is a robust gateway system that could be adapted to the analysis of Alu-mediated homologous recombination in the human BRCA1 locus in breast tumors (6) and other diseases including FA (9,128), or more generally to probe the causes and consequences of DSBs in human cells (129). Combined with knockdowns of specific genes of interest or testing of chemical/medicinal compounds, the dual fluorescence system can quantitatively report on the contribution of specific proteins to HR and NHEJ and is therefore well suited for high throughput systematic studies of Alu-mediated recombination events.

## Supplementary Material

Supplementary Data
