# Supplementary material for: Deficiency of the Fanconi anemia E2 ubiqitin conjugase UBE2T only partially abrogates Alu-mediated recombination in a new model of homology dependent recombination
Source: Nucleic Acids Res. 2019 Feb 1;47(7):3503–20. doi: 10.1093/nar/gkz026 (PMC6468168; doi:10.1093/nar/gkz026)
Supplement: Supplementary Data [file gkz026_supplemental_files.pdf]

Alignment of Sequences: [eGFP ORF.xdna] with Sequence_2: [TOMATO ORF.xdna]

```
Seq_1  1     atggtgagcaagggcgaggag-ctgttcaccggggtggtgcccatcctggtcgagctgga  59
             |||||||||||||||||||||     |||  | | |  ||| | ||  |||     ||||
Seq_2  1     ATGGTGAGCAAGGGCGAGGAGGTCA-TCAAAGAGTTCATGCGCTTCAAGGTGCGCATGGA  59

Seq_1  60    cggcgacgtaaacggccacaagttca-gcgt-gtccggcgagggcgagggcgatgccacc  117
              |||  | | ||||||||| |||||  |  | |   |||||||||||||||
Seq_2  60    GGGCTCCATGAACGGCCACGAGTTCGAGA-TCGAG-GGCGAGGGCGAGGGC---------  108

Seq_1  118   tacggcaagctgaccctgaagttcatctgcaccaccggcaagctgcccgtgccctggccc  177
                      | | |||  | |        ||   |    |           ||  || |
Seq_2  109   ---------C-G-CCCCTACGAGGGCACCCAGACCGCCAAGCTGAAGGTGACCAAGGGCG  157

Seq_1  178   accctcgtgaccaccctgacctacggcgtgcagtgcttcagccgctaccccgaccacatg  237
              ||| |    |  | |          |   |    |       |     |
Seq_2  158   GCCCCCTGCCCTTCGCCTGGGACATCCTGTCCCCCCAGTTCATGTACGGCTCCAAGGCGT  217

Seq_1  238   aagcagcacgacttcttcaagtccgccatgcccgaaggctacgtccaggagcgcaccatc  297
             | |     |  |      |  ||| | ||  |   | |||          | |  |
Seq_2  218   ACGTGAAGCACCCCGCCGACATCCCCGATTACAAGAAGCTGTCCTTCCCCGAGGGCTTCA  277

Seq_1  298   ttcttcaaggacgacggcaactacaagacccgcgccgaggtgaagttcgagggcgacacc  357
                   |  |      | |  |  | |||    | |  |     ||      |     |
Seq_2  278   AGTGGGAGCGCGTGATGAACTTCGAGGACGGCGGTCTGGTGACCGTGACCCAGGACTCCT  337

Seq_1  358   ctggtgaaccgcatcgagctgaagggcatcgacttcaaggaggacggcaacatcctgggg  417
             |     |                    |           | ||     |    ||    |
Seq_2  338   CCCTGCAGGACGGCACGCTGATCTACAAGGTGAAGATGCGCGGCACCAACTTCCCCCCCG  397

Seq_1  418   cacaagctggagtacaactacaacagccacaacgtctatatcatggccgacaagcagaag  477
                   | | | | ||     |           |            |   |      |
Seq_2  398   ACGGCCCCGTAATGCAGAAGAAGACCATGGGCTGGGAGGCCTCCACCGAGCGCCTGTACC  457

Seq_1  478   aacggcatcaaggtgaacttcaagatccgccacaacatcgaggacggcagcgtgcagctc  537
               ||  |    |           ||    |  ||    |     | | ||   |  |  |
Seq_2  458   CCCGCGACGGCGTGCTGAAGGGCGAGATCCACCAGGCCCTGAAGCTGAAGGACGGCGGCC  517

Seq_1  538   gccgaccactaccagcagaacacccccatcggcgacggccccgtgctgctgcccgacaac  597
              |   |   |        |   |  | |   |     |   |  |        |
Seq_2  518   ACTACCTGGTGGAGTTCAAGACCATCTACATGGCCAAGAAGCCCGTGCAACTGCCCGGCT  577

Seq_1  598   cactacctgagcacccagtccgccctgagcaaagaccccaacgagaagcgcgatcacatg  657
                     | | || |    |              | | |    ||             |
Seq_2  578   ACTACTACGTGGACACCAAGCTGGACATCACCTCCCACAACGAGGACTACACCATCGTGG  637

Seq_1  658   gtcctgctggagttcgtgaccgccgccgggatcactct-----cggcatggacgagctgt  712
               |     |        ||  ||||||   | |  |       |||||||||||||||||
Seq_2  638   AACAGTACGAGCGCTCCGAGGGCCGCCACCACCTGTTCCTGTACGGCATGGACGAGCTGT  697

Seq_1  713   acaagtaa  720
             ||||||||
Seq_2  698   ACAAGTAA  705
```

Supplementary Figure 1. Alignment of eGFP and dTomato cDNA sequences.

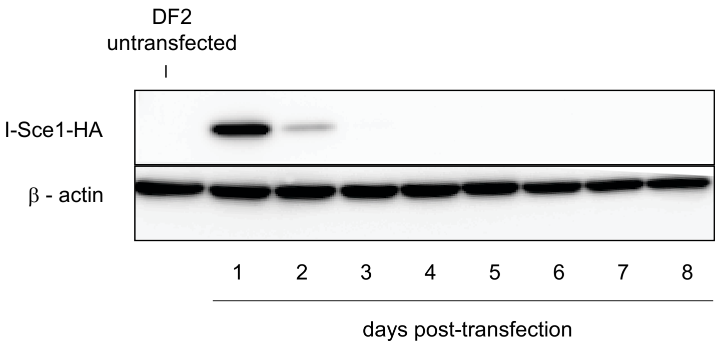

Supplementary Figure 2. I-*Sce1* expression in DF2 cells. DF2 cells were transfected with the vector expressing I-*Sce1* and analyzed for protein expression by western blotting.

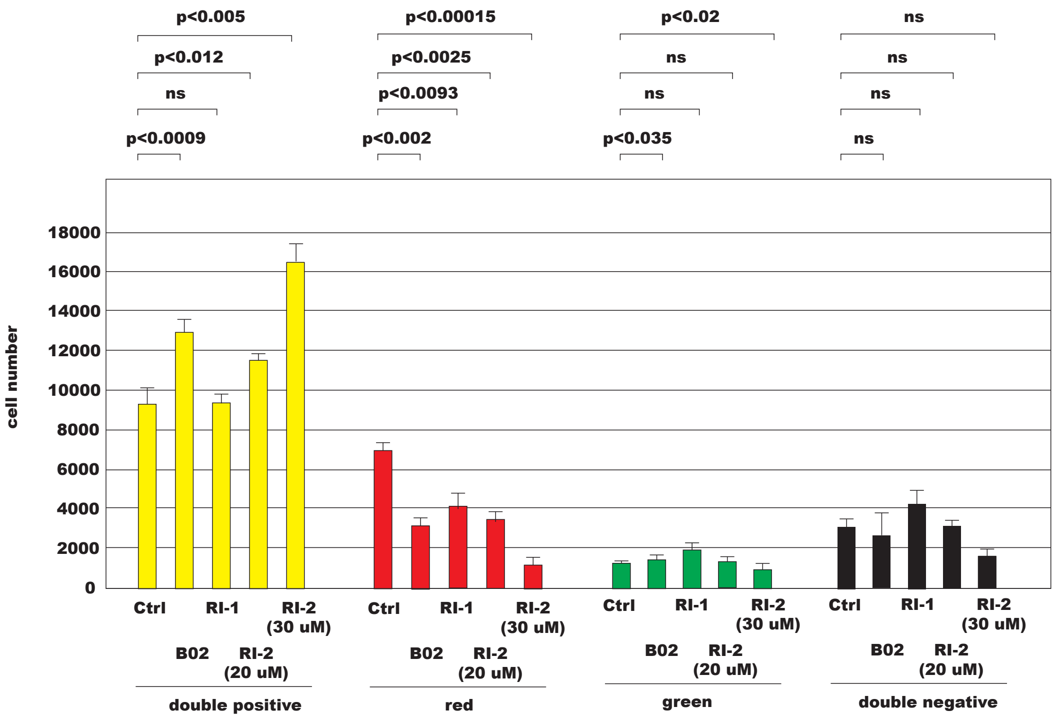

Supplementary Figure 3. Inhibition of Alu-mediated recombination by RAD51 inhibitors. Quantitation of flow cytometry results of DF2 cells treated with I-*Sce1* and RAD51 inhibitors (B02 (105,130), RI-1 (73), RI-2 (99)). Data are the means + S.D. of three biological replicates each assayed in triplicate (paired Student's t-test). Values of p>0.05 were considered not significant (ns).

Supplementary Table 1

FA/HR related protein	GFP positive test cells vs. control	reference
FANCA	~35% [a]	(131)
FANCA	~40% [b]	(132)
FANCA	~50%[d]	(85)
FANCC	~30% [c]	(133)
FANCC	~60%[d]	(85)
FANCD1 (BRCA2)	~20% [d]	(134)
FANCD1 (BRCA2)	~20% [e]	(135)
FANCD2	~100% [e]	(135)
FANCD2	~50% [d]	(134)
FANCD2	~50% [b]	(132)
FANCD2	~50% [d]	(85)
FANCE	~75% [d]	(85)
FANCF	~60% [d]	(85)
FANCG	~90% [d]	(85)
FANCG	~40% [b]	(132)
FANCI	~50% [d]	(134)
FANCI	~90% [d]	(85)
FANCJ (BRIP1, BACH1)	~10% [f]	(136)
FANCJ (BRIP1, BACH1)	~10% [g]	(137)
FANCL	~65% [d]	(85)
FANCM	~40% [g]	(137)
FANCM	~45%	(85)
FANCN (PALB2)	~30% [d]	(138)
FANCO (RAD51C)	~30% [h]	(139)
FANCP (SLX4)	~75% [i]	(140)
FANCS (BRCA1)	~25% [d]	(138)
FANCS (BRCA1)	~20% [j]	(86)
FANCS (BRCA1)	<10% [f]	(136)
FANCS (BRCA1)	<10% [g]	(137)
FANCT (UBE2T)	~60%	this work
FAAP24	~35% [d]	(85)
ATM	~70% [k]	(86)
ATM	~100% [l]	(86)
ATR	~40% [d]	(134)
ATR	~20% [i]	(140)
RAD18	~30% [m]	(139)

Supplementary Table 1. Decrease in DR-GFP homologous recombination in UBE2T$^{-/-}$ cells. Comparison of DR-GFP homologous recombination efficiency in mutant or knockdown cells vs. controls. [a] *$fanca^{\Delta/\Delta}$* vs. wild type mouse fibroblasts; [b] noncomplementing vs. complementing virus in FA cells; [c] ΔFANCC knockout vs. wild type DT40 cells; [d] siRNA knockdown vs. siControl in U2OS cells ; [e] FA cells vs. expression plasmid complemented cells; [f] siRNA knockdown vs. siLuciferase in MCF7 cells; [g] plasmid shRNA knockdown vs. control shRNA in U2OS cells; [h] control vs. complementing virus in C deficient IRS3 cells; [i] viral shRNA knockdown vs. viral control shRNA in U2OS cells; [j] BRCA1$^{tr/tr}$ (truncated) vs. wild type in MEFs; [k] ATM null vs. wild type MEFs; [l] ATM null vs. wild type primary ear fibroblasts; [m] control vs. complementing virus in Rad18$^{-/-}$ MEFs.
